# Performance evaluation of a second‐generation O‐ring‐shaped image‐guided radiotherapy system with a gimbal‐mounted linear accelerator and real‐time tracking capabilities

**DOI:** 10.1002/acm2.70329

**Published:** 2025-11-07

**Authors:** Kohei Kawata, Yukako Kishigami, Hideaki Hirashima, Yohei Sawada, Maika Urago, Takahiro Fujimoto, Tetsuo Fukuda, Takashi Mizowaki, Mitsuhiro Nakamura

**Affiliations:** ^1^ Department of Radiation Oncology and Image‐Applied Therapy Graduate School of Medicine Kyoto University Kyoto Japan; ^2^ Division of Clinical Radiology Service Kyoto University Hospital Kyoto Japan; ^3^ X‐ray Therapy Division, Therapy System Business, Healthcare Business Group Hitachi High‐Tech Corporation Tokyo Japan; ^4^ Department of Advanced Medical Physics Graduate School of Medicine Kyoto University Kyoto Japan

**Keywords:** commissioning, IGRT verification, OXRAY, TPS verification, tracking performance

## Abstract

**Purpose:**

OXRAY, a state‐of‐the‐art radiation therapy system commercialized by Hitachi High‐Tech Ltd. in 2023, integrates unique beam delivery and image‐guided radiation therapy (IGRT) technologies as the successor to Vero4DRT. This study evaluated the performance of this second‐generation O‐ring‐shaped linear accelerator.

**Methods:**

The percentage depth dose (*PDD*) and off‐center ratio (*OCR*) were calculated using the RayStation 2023B treatment planning system with multileaf collimator‐shaped square fields. *PDDs* were evaluated up to a depth of 250 mm and *OCRs* at depths of 15, 100, and 200 mm, compared with measurements. Patient‐specific quality assurance (PSQA) was conducted for 28 volumetric‐modulated arc therapy plans and evaluated using gamma pass rates (GPRs) based on a 3%/2 mm criterion. The biaxial rotational dynamic radiation therapy (BROAD‐RT) performance was validated with 25 trajectories. A tracking experiment under rotational irradiation was performed to assess the tracking accuracy. Additionally, image‐guidance systems (kV X‐ray and kV cone‐beam computed tomography) were evaluated using anthropomorphic phantoms. The localization accuracy (LA) was determined by comparing the known offsets with the noted differences between the initial and corrected positions.

**Results:**

Differences between the calculated and measured data were within the tolerance limits defined in European Society for Radiotherapy and Oncology Booklet 7 and American Association of Physicists in Medicine (AAPM) Medical Physics Practice Guideline 5.b. The median PSQA GPRs exceeded 95%, satisfying AAPM Task Group‐218 criteria. BROAD‐RT demonstrated submillimeter accuracy (within 0.4 mm), even for complex trajectories. The tracking accuracy remained within 1 mm even during rotational delivery. LA was within 0.5 mm for translational shifts and 0.5° for rotational adjustments.

**Conclusion:**

OXRAY demonstrated clinically acceptable beam quality and high‐precision dose delivery outcomes. The tracking accuracy was maintained under rotational irradiation. Automatic image registration enabled accurate, reproducible patient positioning, supporting reliable IGRT implementation. These findings offer practical guidance and technical benchmarks for institutions adopting OXRAY.

## 1 INTRODUCTION

In recent years, rapid advancements in radiation therapy have considerably enhanced cancer treatment outcomes. Among these, the development and clinical integration of innovative treatment systems have played a pivotal role in enhancing dose delivery precision, minimizing treatment‐related toxicities and enabling more individualized therapeutic strategies.

OXRAY, a next‐generation radiation therapy system developed by Hitachi High‐Tech Ltd. in 2023, represents a substantial evolution from its predecessor, Vero4DRT.^1^ While inheriting the core design concept of an O‐ring gantry with a gimbaled X‐ray head for real‐time tumor tracking, OXRAY addresses several of the technical limitations of Vero4DRT—such as the lack of a flattening filter‐free (FFF) beam, relatively coarse multileaf collimator (MLC) resolution, low O‐ring rotation speed, and the inability to perform volumetric modulated arc therapy (VMAT) under real‐time tumor tracking conditions—by incorporating enhanced mechanical and dosimetric capabilities. Notably, it enhances biaxial rotational dynamic radiation therapy (BROAD‐RT)—a noncoplanar dynamic irradiation technique involving the simultaneous motion of the O‐ring and gantry—and newly supports VMAT under real‐time tumor tracking, both of which represent major advancements compared with the previous system.

Following its installation at our institution in 2024, we conducted a commissioning process to ensure the system's clinical readiness. While commissioning is a standard practice in radiotherapy,^2^, ^3^ the distinctive mechanical and dosimetric characteristics of OXRAY, particularly those related to BROAD‐RT and gimbaled tracking, require tailored approaches that go beyond conventional protocols.^4^, ^5^ In this context, there is a pressing need to develop and validate quality assurance (QA) and treatment verification methodologies that are specifically optimized for the complex motion geometries and real‐time capabilities unique to OXRAY.

This study presents an initial evaluation of the dosimetric and image‐guidance performance of the OXRAY system, with a specific focus on the geometric and dosimetric verification of BROAD‐RT. We also conducted a detailed investigation of VMAT under real‐time tumor tracking conditions, including its integration with BROAD‐RT, to assess the feasibility and accuracy of this novel combination. A novel QA methodology using the system integrated electronic portal imaging device (EPID) was implemented to assess beam trajectory accuracy during dynamic delivery. This approach not only ensures safe clinical implementation but also represents a methodological contribution toward exploiting the full potential of OXRAY's unique features. Our findings aim to provide extra knowledge for future development of treatment strategies and QA procedures optimized for next‐generation O‐ring linear accelerators.

## 2 MATERIALS AND METHODS

### 2.1 System description for beam delivery system

**FIGURE 1:**
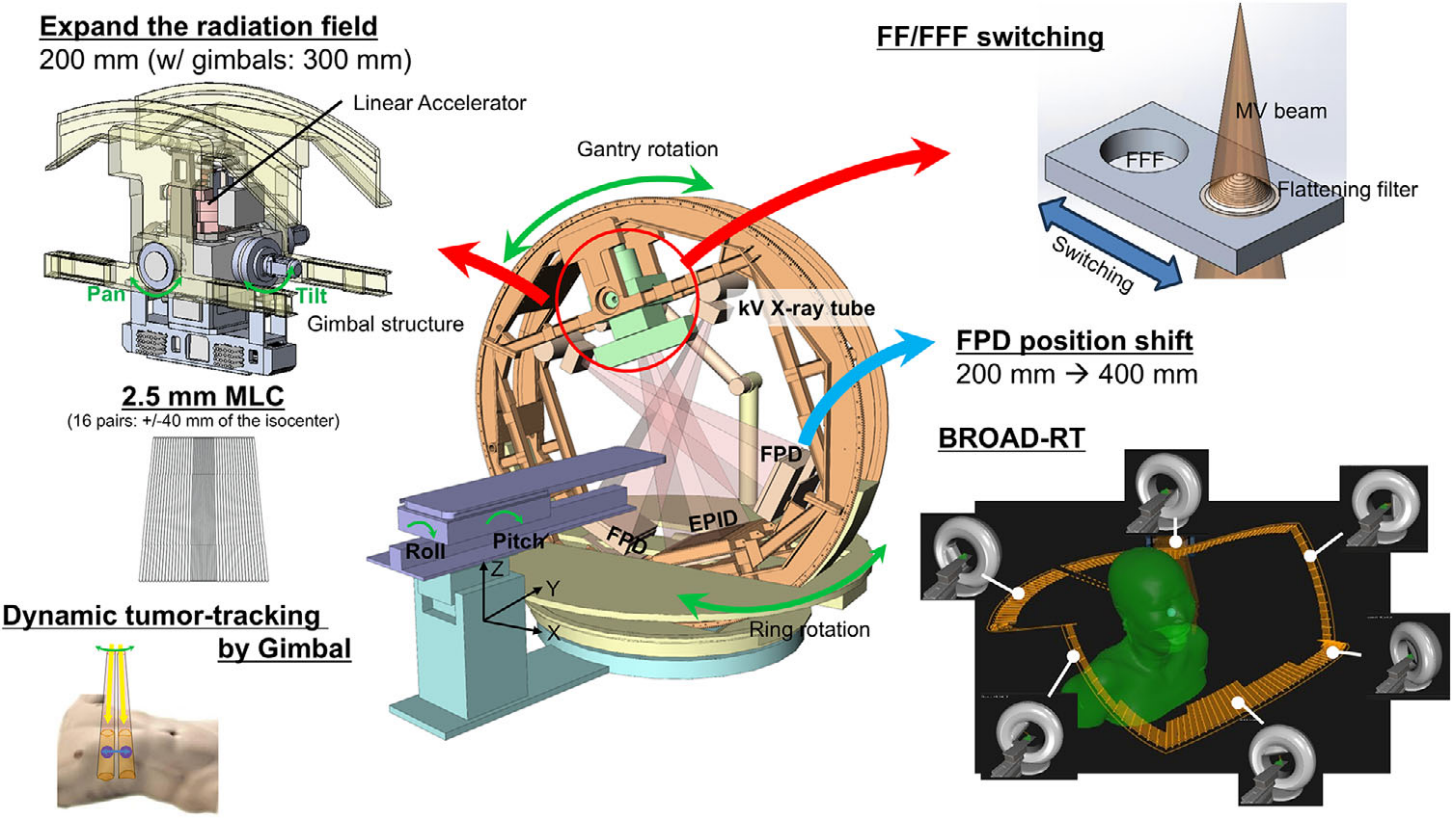
Overview of the OXRAY system and its key capabilities. The figure illustrates the system configuration, including the dual kV imaging units and O‐ring gantry, as well as its core functionalities, such as expansion of the radiation field by the gimbaled head, flattening filter (FF)/flattening‐filter‐free (FFF) switching, real‐time tumor tracking with volumetric modulated arc therapy and biaxial rotational dynamic radiation therapy (BROAD‐RT). This schematic provides a comprehensive summary of the technological innovations implemented in the OXRAY system.

Figure 1 shows a schematic of the OXRAY system. OXRAY features an O‐ring‐shaped structure and a gimbaled head, enabling pan and tilt rotations. The gantry rotates within the ring at a maximum speed of 7°/s, enabling a rotational range of ± 185°. In addition, the O‐ring itself can rotate around its vertical axis at a maximum speed of 6°/s within a range of ± 60°. The gimbaled head is equipped with two orthogonal gimbals that enable independent pan and tilt rotations of up to ± 3°, with a maximum rotational speed of 6°/s. This rotational capability allows the radiation beam to be dynamically shifted by up to ± 50.3 mm in each direction on the isocenter plane. The system offers 6 MV and 6 MV FFF X‐ray beam energies, with maximum dose rates of 600 and 1200 Monitor unit (MU)/min, respectively.

The treatment aperture features an MLC with a 2.5 mm leaf width for the inner 40 mm field, while the remaining MLC leaves are 5 mm wide. The MLC is made from a tungsten alloy with 95% purity. The leakage between adjacent leaves is minimized by an interlocking tongue‐and‐groove arrangement, with the height of the groove part being 55 mm. The MLC has a height of 11 cm and length of 26 cm and operates at a maximum speed of 65 mm/s at the isocenter. Each leaf end is circular, with a radius of curvature of 370 mm.^6^ The secondary collimator in the OXRAY system is of a fixed type, with the aperture formed exclusively by the MLC. The maximum field size is 200 × 200 mm^2^. The collimator does not include a rotational mechanism.

A distinctive characteristic of the OXRAY system is its implementation of BROAD‐RT. In BROAD‐RT, both the gantry and the O‐ring are simultaneously rotated around two different axes, facilitating noncoplanar VMAT without the need for patient movement. This technology includes a customizable trajectory, with notable capabilities.^7^, ^8^, ^9^

The EPID system has a pixel area of 19.8 × 19.8  cm^2^ at the isocenter and provides a spatial resolution of 0.13 mm at the isocenter using 1536 × 1536 pixels with a 16‐bit depth.

#### 2.1.1 Beam data acquisition and beam modeling

A three‐dimensional (3D) water phantom (Blue Phantom SMARTSCAN, IBA Dosimetry, Schwarzenbruck, Germany) was used for both scanning and nonscanning data acquisitions of the percentage depth dose (*PDD*), off‐center ratio (*OCR*), and output factor (*OPF*). All measurements were performed at a source‐to‐surface distance of 100 cm. Ionization chambers (CC04 and CC01, IBA Dosimetry, Schwarzenbruck, Germany) were used for field sizes ≥ 40 × 40 mm^2^ and < 40 × 40 mm^2^, respectively. *OPF* measurements were conducted at a depth of 100 mm for square and rectangular fields ranging from 20 × 20 to 200 × 200 mm^2^, with the 100 × 100  mm^2^ field defined as the reference field. The *PDD*, *OCR*, and *OPF* required for beam modeling in the RayStation 2023B (RaySearch Laboratories, Stockholm, Sweden) treatment planning system (TPS) were thus acquired.

The beam data were registered in the RayStation 2023B TPS, and beam modeling was initiated by registering the measured *PDD, OCR*, and *OPF* data. The curve quality in RayPhysics was employed for evaluation of the agreement. For *PDD* data, the curve quality was assessed for both the build‐up region and the fall‐off region. For *OCR* data, the curve quality was computed for the in‐field, penumbra and out‐of‐field region. The curve quality value is the root mean square (RMS) difference between measured data and computed data. The initial MLC parameters were set such that the RMS difference was within 0.5% for all field sizes in the *PDD*, and within 1% for all field sizes and all depths in the *OCR*. Subsequently, the MLC parameters were tuned using the four VMAT quality assurance (QA) plans, including the C‐shape (*n* = 1, coplanar VMAT), brain (*n* = 1, BROAD‐RT), lung (*n* = 1, noncoplanar VMAT), and prostate (*n* = 1, BROAD‐RT). The C‐shape is a benchmark plan used at our institution, and the other plans were selected to represent anatomical sites that are expected to be frequently treated with the OXRAY system. In addition, a previous study has suggested that BROAD‐RT plans include multiple manipulation points, which may negatively affect the dosimetric robustness.^10^ Therefore, in this study, we decided to perform verification using all irradiation techniques: coplanar VMAT, noncoplanar VMAT, and BROAD‐RT. The final MLC parameters met the tolerance or action levels specified by the American Association of Physicists in Medicine Task Group No. 218 (AAPM TG‐218).^11^ ArcCHECK (Sun Nuclear Corporation, Melbourne, FL, USA) was used to measure the dose distributions of the plans. Gamma pass rate (GPR) analysis was conducted for the QA plans based on a 3%/2 mm criterion with a low‐dose threshold of 10% ^10^ by using the SNC patient software program (version 8.6.0, Sun Nuclear Corporation, Melbourne, FL, USA). All treatment plans were generated using the RayStation 2023B TPS with the collapsed cone convolution (CCC) algorithm (version 5.8) and a calculation grid size of 2 mm. The density of ArcCHECK was set to 1.15 g/cm^3^ in the TPS.

After the MLC parameters were fixed, *PDD* and *OCR* calculations were performed using the CCC algorithm in the TPS with a calculation grid size of 1.0 mm. The calculations were conducted using the MLC‐shaped square fields of 20 × 20, 50 × 50, 100 × 100, and 200 × 200 mm^2^. The *PDD* was calculated at depths of up to 250 mm, while the *OCR* was calculated at depths of 15, 100, and 200 mm. The comparison of the calculated and measured data, converted to cGy/MU values, was performed according to the criteria set in the European Society for Radiotherapy and Oncology (ESTRO) Booklet 7^12^ based on the low‐dose‐gradient area beyond *d_max_* ($\delta_{1}$), the buildup or penumbra region ($\delta_{2}$), the low‐dose‐gradient area inside the beam but off the central axis ($\delta_{3}$), the low‐dose‐gradient area outside the geometrical beam ($\delta_{4}$), and AAPM's Medical Physics Practice Guideline 5.b ^13^ based on the high‐dose, penumbra, and low‐dose tails.

#### 2.1.2 Patient‐specific QA

Subsequently, patient‐specific QA (PSQA) was performed using ArcCHECK. In total, 10 VMAT plans (coplanar = 2, noncoplanar = 2, BROAD‐RT = 6) were evaluated using 6 MV photon beams for various treatment sites, including the brain (*n *= 3), lungs (*n *= 3), head and neck (*n *= 2), and prostate (*n *= 2). Additionally, 18  VMAT plans (coplanar = 2, noncoplanar = 10, BROAD‐RT = 6) were analyzed using 6 MV‐FFF photon beams for treatment sites, including the brain (*n *= 1; large planning target volume (PTV), *n *= 5; small PTV; PTV size: 6.92–12.37 cm^3^), lung (*n *= 4), head and neck (*n *= 2), prostate (*n *= 2), liver (*n *= 1), pancreas (*n *= 1), and spine (*n *= 2). These plans were selected independently from those used to determine the initial parameters, and representative patients were chosen for each anatomical site that is a potential target for treatment with each output energy of the OXRAY system. All treatment plans were generated using the RayStation 2023B TPS with the CCC algorithm. In brain cases with small PTV volumes, for which noncoplanar VMAT plans were created, per‐arc dose measurements were conducted to avoid beam delivery to the ArcCHECK circuitry. Consequently, 28 PSQA measurements were performed for the 6 MV‐FFF plans. GPR analysis was performed as described above. If the GPR fell below 90%, film dosimetry was performed using Gafchromic EBT4 films (Ashland Inc., Bridgewater, NJ, USA).

### 2.2 BROAD‐RT performance

**FIGURE 2:**
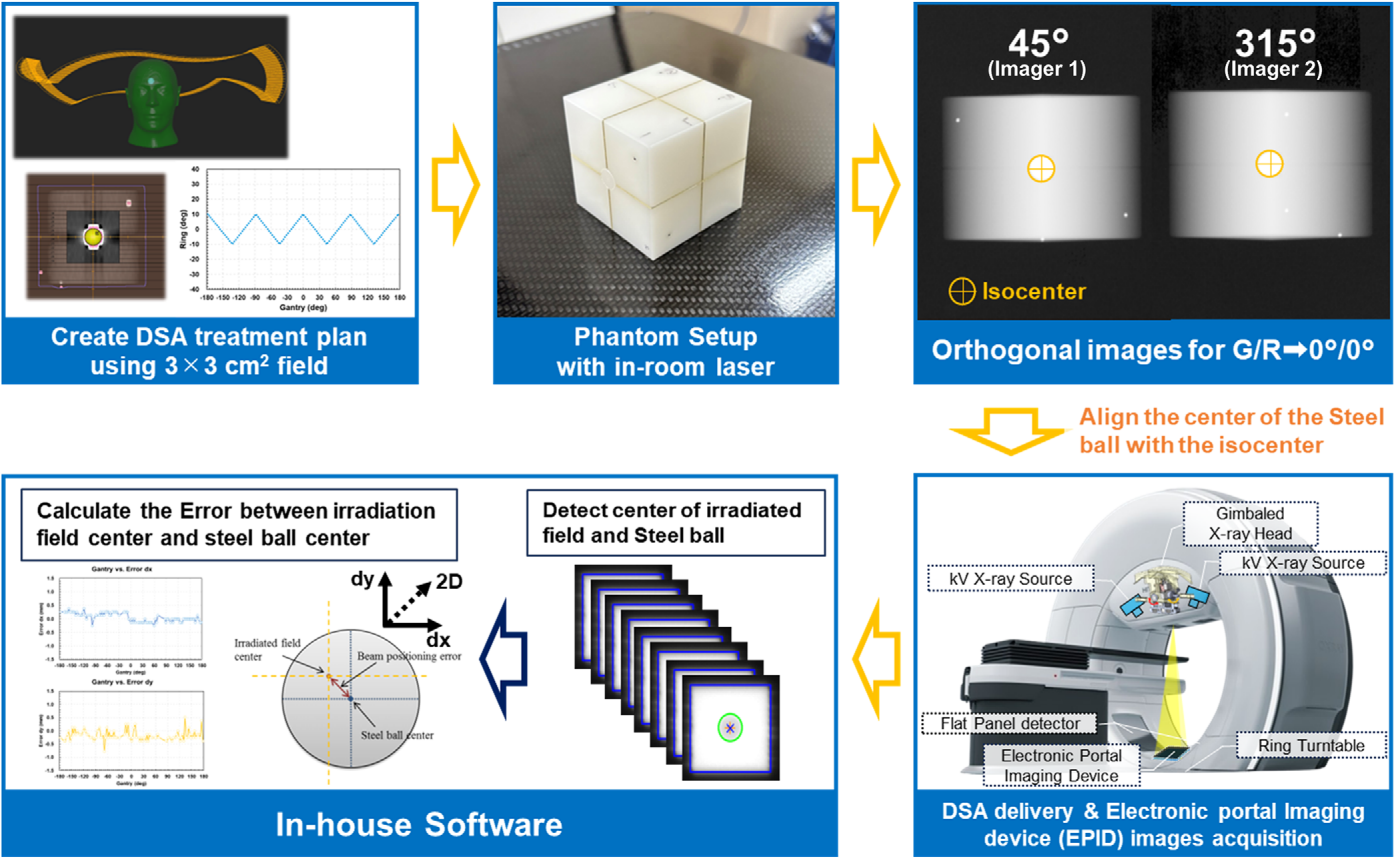
Validation summary of the BROAD‐RT performance.

**FIGURE 3:**
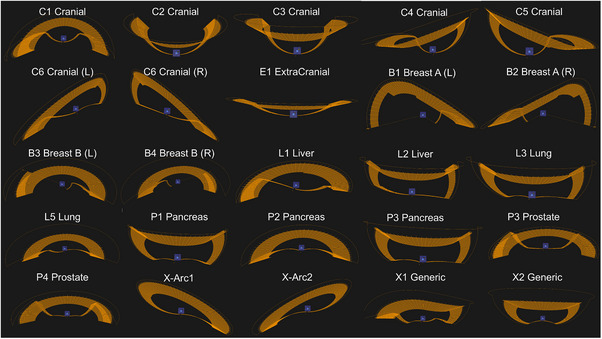
Arc trajectories of BROAD‐RT. Refer to Table S4 for the deliverable manipulation point (Ring/Gantry angles) for each trajectory.

Figure 2 illustrates the validation methodology employed to evaluate the BROAD‐RT performance of OXRAY. A cube‐shaped phantom containing a centrally embedded steel ball was used as the measurement object. Among the available beam trajectories in BROAD‐RT, 25 representative arc trajectories implemented in RayStation 2023B were selected for analysis (Figure 3). These presented arc trajectories are available as templates in the RayStation 2023B TPS and can be used clinically. These trajectories can also be edited if necessary. The isocenter was set at the center of the steel ball on the CT images, and BROAD‐RT plans were generated using a 6 MV X‐ray beam with a field size of 3 × 3 cm^2^ and a monitor unit (MU) setting of 600. Following the initial positioning of the phantom using in‐room lasers, orthogonal kV X‐ray images were acquired to refine alignment. The phantom was adjusted such that the steel ball center coincided with the planned isocenter. Irradiation was then performed, during which EPID images were acquired in synchrony with beam delivery. For each trajectory, experiments were performed three times to ensure reproducibility and robustness of the evaluation. To assess the geometric accuracy, the centers of the steel ball and radiation field were identified on each EPID image using a standalone in‐house software tool developed specifically for this study.^14^ This software is not integrated into the OXRAY system and independently processes EPID images using threshold‐based image analysis, referencing the known dimensions of the radiation field and steel ball. The spatial discrepancy between the two centers was calculated and used as a metric of beam positioning error. For analysis, EPID images affected by beam interference because of the treatment couch were excluded. As a result, approximately 100 EPID images per trajectory were analyzed, corresponding to acquisition intervals of every 3° of the gantry rotation during delivery.

### 2.3 Tracking performance

**FIGURE 4:**
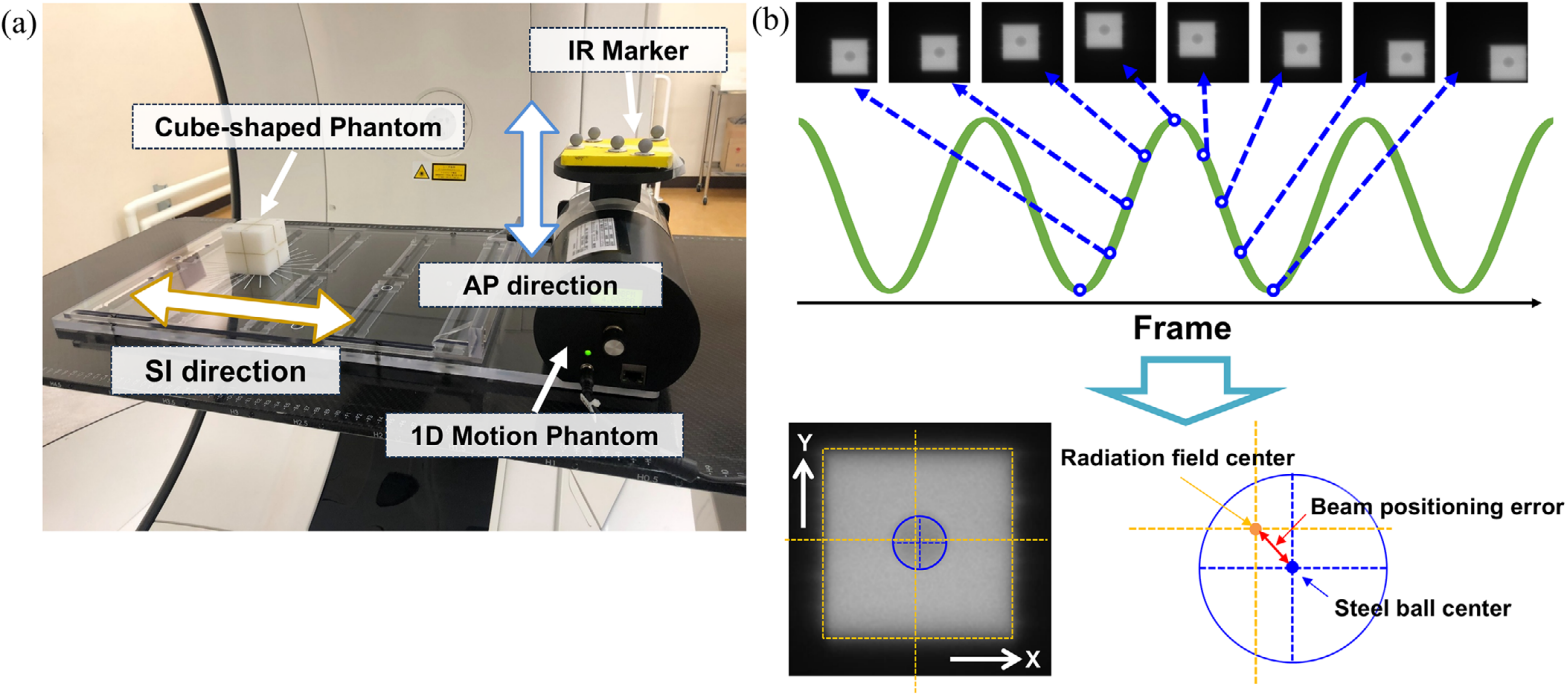
Experimental setup used to investigate the tracking performance (a). The beam positioning error was defined as the difference between the centers of the steel ball and radiation field using electronic portal imaging device images during infrared reflective (IR) tracking (b).

The QUASAR Programmable Respiratory Motion Platform (Modus Medical Device Inc., London, ON, Canada), which enables the synchronized motion of phantoms with external respiratory surrogates, was employed in this study. Infrared reflective (IR) markers were used as external surrogates, and they oscillated in the anterior–posterior (AP) direction. Simultaneously, a cube‐shaped phantom, simulating a tumor with a central steel ball and radiopaque fiducial markers implanted peripherally, was driven in the superior–inferior (SI) direction according to a one‐dimensional sinusoidal motion pattern (amplitude: ± 20 mm; cycle: 6 s), synchronized with the IR markers. There was a one‐to‐one correspondence between the phantom's motion in the SI direction and the IR markers' motion in the AP direction. (Figure 4(a)).

Before beam delivery, the IR markers and the implanted fiducial markers were tracked simultaneously over a 20 s period using an IR camera and an orthogonal kilovoltage (kV) X‐ray imaging system, respectively. The sampling interval was 16.7 ms for the IR camera, while the orthogonal kV imaging system acquired data at intervals of either 320 or 640 ms, depending on the speed of the IR marker motion, as it automatically adjusted its sampling rate accordingly. Based on the recorded training data, a correlation model (four‐dimensional (4D) model) was constructed to estimate the three‐dimensional (3D) target position from the IR marker position on the abdominal surface. The model was formulated as a quadratic function incorporating both the IR marker position and its velocity.^15^, ^16^

The radiation field was set to 3 × 3 cm^2^, and the gantry was rotated through 360°. A 6 MV X‐ray beam was delivered with an MU setting of 500. The experiment was conducted five times at each of the ring angles of 0°, 20°, and 340°. The analysis method using the software was the same as that described in Section 2.2. For the analysis, EPID images affected by the interference induced owing to the transmission of the beam through the couch were excluded. Consequently, approximately 100 EPID images were analyzed at various gantry angle settings (every 3°) during beam delivery. The beam positioning error was calculated as the distance between the radiation field center and steel ball center (Figure 4(b)). As part of an ablation study, we also investigated the combined implementation of BROAD‐RT and real‐time tumor tracking to assess its feasibility and geometric accuracy. Specifically, six trajectories—L1 Liver, L2 Liver, L3 Lung, L5 Lung, P1 Pancreas, and P2 Pancreas—were selected from those illustrated in Figure 3. These trajectories were chosen based on their clinical relevance, as they are designed for anatomical sites subject to respiratory‐induced motion. For each trajectory, experiments were performed three times to ensure the reproducibility and robustness of the evaluation. This selection and experimental design allowed us to assess the tracking performance under conditions that closely emulated actual treatment scenarios involving moving targets.

### 2.4 Validation of image guidance system

#### 2.4.1 System description for IGRT system

An orthogonal pair of kV X‐ray imaging systems is integrated into the O‐ring gantry at ± 45° relative to the MV beam axis, enabling the simultaneous acquisition of two orthogonal kV X‐ray images as well as dual‐source kV cone‐beam computed tomography (kV‐CBCT) images. The imaging system is rotated synchronously with the gantry and O‐ring structures. The flat panel detector (FPD) has a pixel area of 21 × 21 cm^2^ and a spatial resolution of 0.15 mm at the isocenter (using 1440 × 1440 pixels) with a 16‐bit depth. The size of the gimbaled head becomes larger as a function of the radiation field. Therefore, the source‐to‐isocenter distance of the kV X‐rays was increased to 105.97 cm to avoid interference between the kV X‐ray beam and the gimbaled head. Consequently, the distance between the source and FPD was set to 208.47 cm, which yielded a CBCT field of view (FOV) of 21 (in the O‐ring plane) × 21 cm^2^ (perpendicular to the O‐ring plane). The FPD is movable; hence, a maximum CBCT FOV of 40 (in the O‐ring plane) × 21 cm^2^ (perpendicular to the O‐ring plane) can be achieved by shifting the FPD away from the axis of the kV X‐ray beam. In dual‐source kV‐CBCT, alternating imaging was performed using Imagers #1 and #2. The imaging time for dual‐source kV‐CBCT is approximately 15 s. The reconstruction algorithms included the Feldkamp and iterative methods, with filtering performed using a proprietary function. Table 1 summarizes the image guidance modes available in the X‐ray and kV‐CBCT systems.

**TABLE 1:** Overview of the image guidance modes available in X‐ray and kV cone‐beam computed tomography systems. *Abbreviations*: FOV, field of view; CBCT, cone‐beam computed tomography; CW, clockwise; CCW, counter‐clockwise.

System	Imaging direction		FOV size	Gantry rotation (Scan time)
kV X‐ray	Oblique	315° and 45°	–	–
	True lateral	0° and 90°, 270° and 0°	–	–
kV‐CBCT	Single (Imagers #1 or #2)	CW, CCW	210 mm in diameter; FOV‐Standard (S)	200° (29 s)
			400 mm in diameter; FOV‐Large (L)	360° (53 s)
	Dual (Imagers #1 and #2)	CW, CCW	210 mm in diameter; FOV‐Standard (S)	116° (15 s)

OXRAY integrates a patient positioning function via a patient positioning image analysis system (PIAS) (Hitachi, Ltd., Tokyo, Japan). In the PIAS registration software program within OXRAY, two types of image registrations were performed for patient setup correction: (1) orthogonal two‐dimensional kV X‐ray images were rigidly registered with the corresponding digitally reconstructed radiograph (DRR) generated from the planning CT (pCT) images, and (2) kV‐CBCT images were rigidly registered with pCT images. For kV X‐ray image registration, alignment was achieved by the zero‐mean normalized cross‐correlation between the X‐ray image and the DRR images. For kV‐CBCT image registration, 3D image registration was performed by optimizing the alignment between the CBCT and pCT images using normalized mutual information, accounting for both translational and rotational adjustments. For kV X‐ray image registration, a region of interest can be defined to mask specific areas; by contrast, for kV‐CBCT image registration, the volume of interest can be designated to constrain the registration area. These settings affected the outcomes of the automatic image registration process; therefore, they were not used in this study. Based on the registration results, the patient's position was corrected using six degrees of freedom. The extent of the patient setup correction was monitored continuously using an IR camera system.

#### 2.4.2 Localization accuracy (LA) by image registration

**FIGURE 5:**
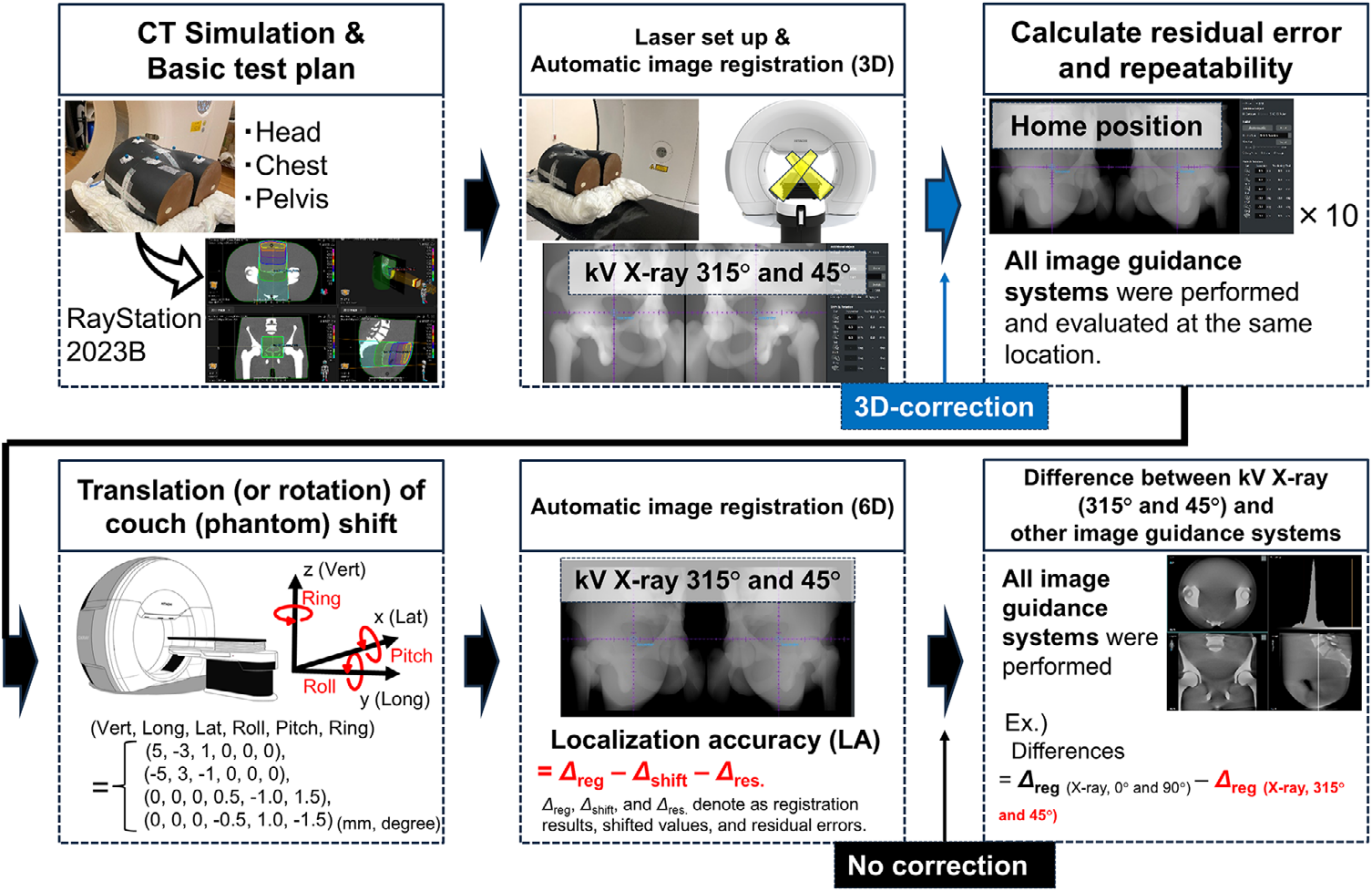
Validation summary of the image‐guidance systems.

Figure 5 illustrates the LA verification process completed via image registration. The LA was evaluated using the PIAS image registration software and anthropomorphic phantoms representing the head, chest, and pelvis. pCT images of the phantoms were acquired using a SOMATOM Definition AS CT scanner (Siemens Medical Systems, Erlangen, Germany) with the following parameters: slice thickness = 2 mm, FOV = 500 mm, and pixel size = 0.98 mm. The acquired CT images were imported into the RayStation 2023B TPS, and basic test plans were generated for each phantom. Each phantom was initially positioned at the isocenter using laser alignment. Subsequently, oblique kV X‐ray images were acquired at paired angles. Automatic image registration was then performed between the kV X‐ray images and the DRRs. Based on the registration results, the position of the phantom was adjusted solely based on couch translation. The corrected position of the phantom was saved as the home position.

At the home position, images were acquired for all image guidance modes listed in Table S1, based on the conditions recommended in the OXRAY instruction manual. The automatic image registration process was repeated 10 times to assess repeatability. The average of the registration results was defined as the residual error, and the standard deviation (SD) of the registration results was calculated to evaluate the repeatability of the image registration process.

Subsequently, an experiment was conducted to evaluate the correction capability. The treatment couch was shifted from the home position according to four predefined patterns: (Vert, Long, Lat, Roll, Pitch, Ring) = (5, ‐3, 1, 0, 0, 0), (‐5, 3, ‐1, 0, 0, 0), (0, 0, 0, 0.5, ‐1, 1.5), and (0, 0, 0, ‐0.5, 1, ‐1.5), where Vert (mm), Long (mm), and Lat (mm) correspond to translations in the vertical, longitudinal, and lateral directions, respectively, and Roll (°), Pitch (°), and Ring (°) to rotations with respect to the lateral, longitudinal, and vertical axes, respectively. Notably, the Ring rotation corresponds to yaw. These shifts were monitored using an IR camera system. Localization images were acquired at each shifted position under the conditions specified in Table S1 and subsequently fused with the planning images to calculate the displacement from the home position. The LA for each image guidance system was calculated as follows:

(1) $$\mathrm{Localization}\mathrm{accuracy}\left(\mathrm{LA}\right)=\Delta_{\mathrm{reg}}-\Delta_{\mathrm{shift}}-\Delta_{\mathrm{res}},$$

where *Δ*_reg_, *Δ*_shift_, and *Δ*_res._ denote the registration results, shifted values, and residual errors, respectively. The Steel–Dwass test was used to analyze the LA, considering the results of all image guidance systems among the head, chest, and pelvis phantoms. The statistical significance was set at *p* < 0.05.

Additionally, various imaging guidance systems can be used in the OXRAY system; however, large differences in the registration results across these systems can be problematic. The registration results of the kV X‐ray images acquired at paired oblique angles (315° and 45°) were used as reference values. The differences in the registration results obtained from kV X‐ray images at paired true lateral angles (0° and 90°, 270° and 0°) and kV‐CBCT images (single‐ and dual‐source; FOV‐S and ‐L; clockwise and counter‐clockwise directions) were then calculated relative to the reference values.

## 3 RESULTS

The MLC parameters registered in the RayStation 2023B TPS were initially determined as follows: tongue‐and‐groove, 0.03 cm; leaf‐tip width, 0.500 cm; offset of MLC position, 0.070 cm; leaf‐tip‐transmission, 0.07 (6 MV) and 0.06 (6 MV‐FFF). The final MLC parameters were determined by adjusting the leaf‐tip width to 0.001 cm. Alterations to other parameters had no impact on the GPR. The GPRs during MLC parameter tuning are summarized in Table 2.

**TABLE 2:** Gamma pass rates for all MLC parameters.

			GPRs at the 3%/2 mm criterion (%)		
			Leaf‐tip width (cm)		
Energy	Treatment site	Treatment technique	0.500	0.005	0.001
6 MV	C‐shape (*n *= 1)	Coplanar	98.2	99.6	99.6
	Brain (*n *= 1)	BROAD‐RT	82.7	96.0	96.1
	Lung (*n *= 1)	Noncoplanar	94.7	94.4	99.0
	Prostate (*n *= 1)	BROAD‐RT	84.4	96.3	96.5
6 MV‐FFF	C‐shape (*n *= 1)	Coplanar	94.9	95.5	95.4
	Brain (*n *= 1)	BROAD‐RT	84.7	94.8	94.8
	Lung (*n *= 1)	Noncoplanar	95.7	98.0	97.7
	Prostate (*n *= 1)	BROAD‐RT	83.5	92.5	92.4

Abbreviations: BROAD‐RT, biaxial rotational dynamic radiation therapy; FFF, flattening‐filter‐free; GPR, gamma pass rate; MLC, multileaf collimator.

**FIGURE 6:**
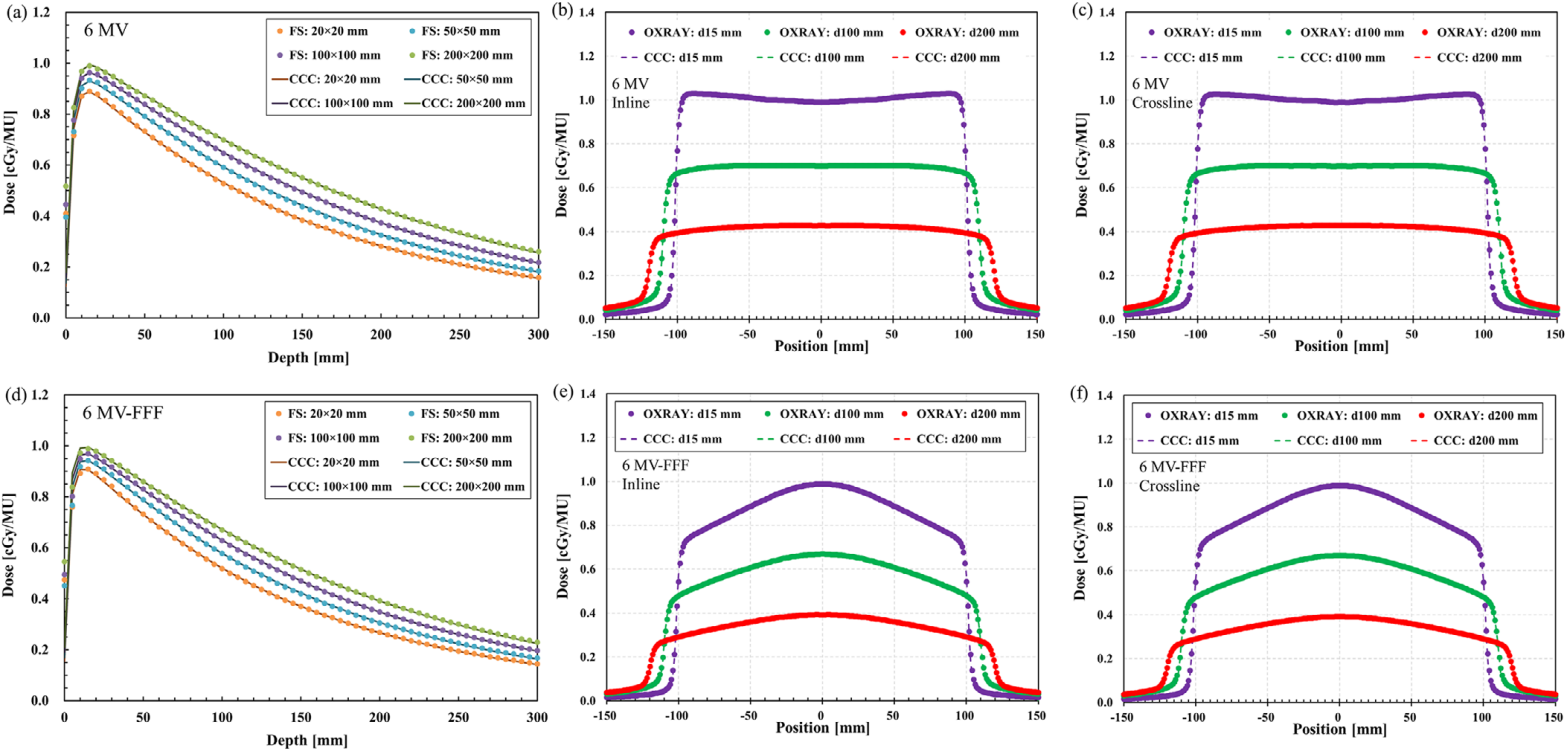
Measured and calculated (a, d) absolute depth dose profiles for all tested field sizes and representative off‐center ratio profiles in the (b, e) inline and (c, f) crossline directions for a 200 × 200 mm^2^ field at depths of 15, 100, and 200 mm using 6 MV (upper) and 6 MV FFF X‐ray beam energies (lower).

Figure 6 illustrates the absolute depth dose profiles for all field sizes and representative *OCR* profiles for a 200 × 200 mm^2^ field with 6 MV and 6 MV‐FFF beams. The maximum values of $\delta_{1}$, $\;\delta_{2}$, $\delta_{3}$, and $\delta_{4}$ are summarized in Tables S2 and S3. Regarding absolute depth‐dose measurements, the $\delta_{1}$ and $\delta_{2}$ values for all field sizes for 6 MV were within 0.8% and 0.7 mm, respectively, and for 6 MV‐FFF, they were within 0.6% and 1.1 mm, respectively. Regarding the *OCR* evaluations, the $\delta_{2}$, $\delta_{3}$, and $\delta_{4}$ values for all field sizes and depths for 6 MV were within 0.3 mm, 1.4%, and 2.3%, respectively, and for 6 MV‐FFF, they were within 0.3 mm, 1.2%, and 1.4%, respectively.

**FIGURE 7:**
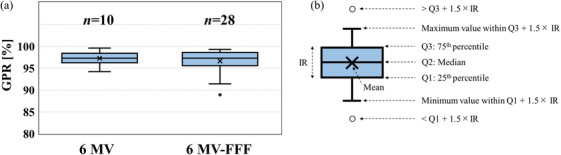
Boxplot of the gamma pass rates (GPRs) based on the 3%/2 mm criterion of for the (a) 6 MV (left) and 6 MV‐FFF plans (right). (b) Interpretation of the boxplot findings.

**FIGURE 8:**
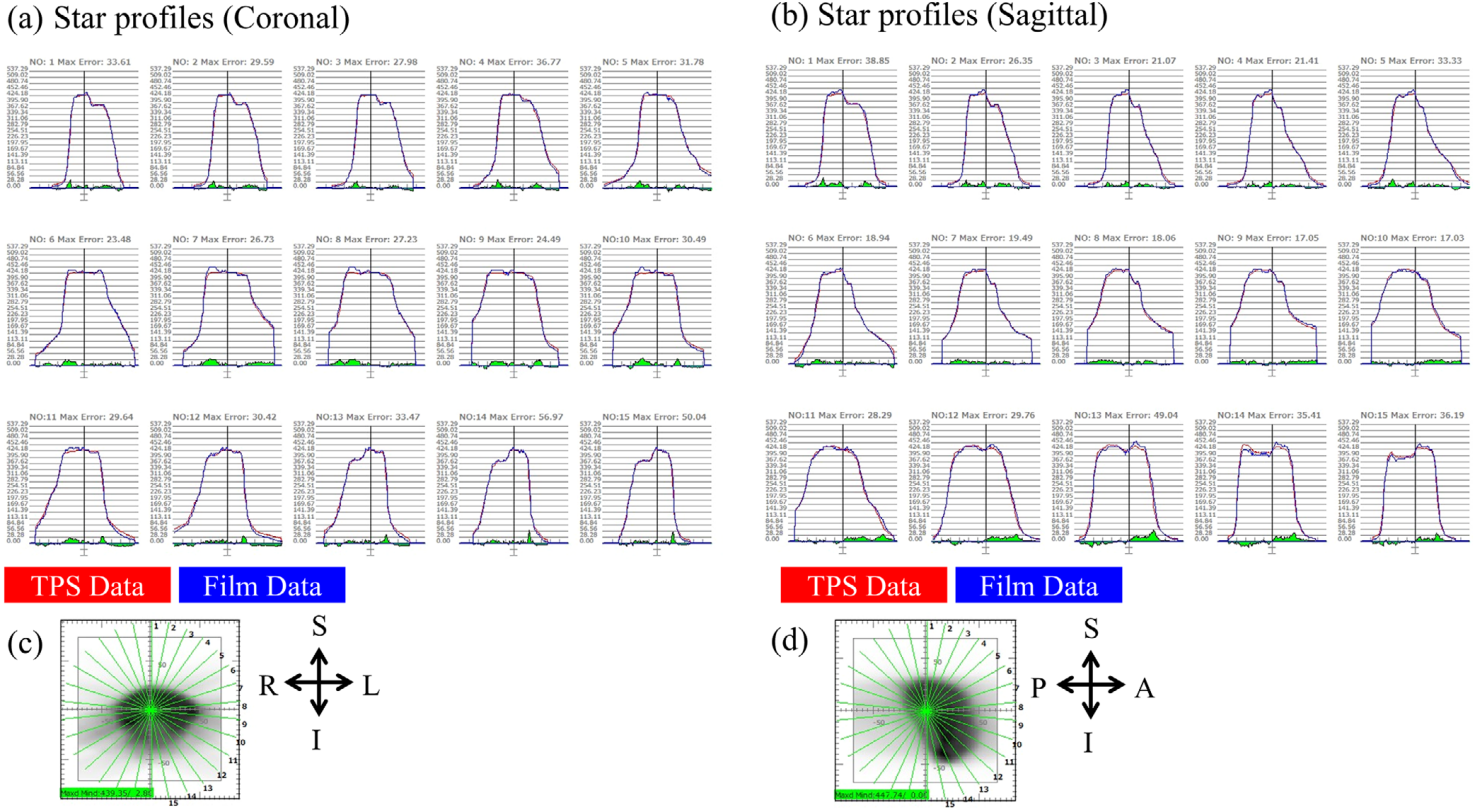
Star profiles for the coronal (a) and sagittal (b) planes along the green lines in (c) and (d). In each profile, the vertical axis represents the dose (cGy).

Figure 7 presents boxplots of the GPRs based on the 3%/2 mm criterion for the 6 MV and 6 MV‐FFF plans across all treatment sites. The median (interquartile range) values of the GPRs were 97.3% (96.3%–98.4%) for the 6 MV plans and 97.3% (95.6%–98.6%) for the 6 MV‐FFF plans. For nine of the ten PSQA measurements at 6 MV, the GPRs were greater than 95%. Similarly, for 23 of the 28 PSQA measurements for the 6 MV‐FFF, the GPRs were greater than 95%. Additionally, for one case in which BROAD‐RT was used, the GPR values were below 90% in the plans using 6 MV‐FFF (88.9%). Given this outlier, additional verification using film dosimetry was performed to further assess the dose‐distribution accuracy in this case. Additional analysis of this case using film dosimetry yielded GPR values of 96.2% and 98.0% (3%/2 mm) for the coronal and sagittal planes, respectively (Figure 8).

Table 3 summarizes the validation for the BROAD‐RT performance of OXRAY using 25 trajectories. Regardless of whether the trajectory was a BROAD‐RT trajectory or not, the mean beam positioning error, defined as the distance between the center of the radiation field obtained from the image and that of the steel ball, was within 0.4 mm.

**TABLE 3:** Summary of the validation for BROAD‐RT performance using 25 trajectories.

BROAD‐RT Trajectory	Beam positioning error (mm)	BROAD‐RT Trajectory	Beam positioning error (mm)
C1 Cranial	0.31 ± 0.12	L1 Liver	0.27 ± 0.10
C2 Cranial	0.29 ± 0.12	L2 Liver	0.28 ± 0.12
C3 Cranial	0.31 ± 0.10	L3 Lung	0.31 ± 0.10
C4 Cranial	0.30 ± 0.12	L5 Lung	0.31 ± 0.11
C5 Cranial	0.31 ± 0.12	P1 Pancreas	0.30 ± 0.11
C6 Cranial (L)	0.32 ± 0.12	P2 Pancreas	0.29 ± 0.10
C6 Cranial (R)	0.37 ± 0.14	P3 Pancreas	0.29 ± 0.11
E1 ExtraCranial	0.27 ± 0.09	P3 Prostate	0.30 ± 0.10
B1 Breast A (L)	0.30 ± 0.14	P4 Prostate	0.30 ± 0.09
B2 Breast A (R)	0.34 ± 0.12	X‐Arc1	0.29 ± 0.11
B3 Breast B (L)	0.30 ± 0.11	X‐Arc2	0.35 ± 0.12
B4 Breast B (R)	0.33 ± 0.11	X1 Generic	0.30 ± 0.10
		X2 Generic	0.29 ± 0.12

The mean ± SD value of the difference between the center of the radiation field obtained from the image and that of the tracked target object was 0.42 ± 0.24 mm during rotational irradiation under the real‐time tumor tracking condition, regardless of the ring rotation angle. Consistently high tracking accuracies were also observed in the ablation study combining BROAD‐RT with real‐time tumor tracking, with the mean differences ranging approximately from 0.42 to 0.48 mm across all six selected trajectories tested three times, and the delivery time ranged from 119 to 178 seconds. Notably, the standard deviations remained comparable to those observed under the standard tracking conditions, indicating that the integration of BROAD‐RT did not compromise the geometric stability or accuracy, even in anatomically dynamic settings.

The automatic image registration of pCT with kV X‐ray and kV‐CBCT at the home position for all phantoms demonstrated excellent repeatability, with SD values of 0.0 mm and 0.0° in all directions. The LA values obtained through image registration (summarized in Table S5) represent the mean ± SD of the LA obtained across four shifted patterns for each phantom and image guidance system. For the anthropomorphic phantoms, the LA was within 0.5 mm and 0.5° in all directions. The differences in the registration results between the kV X‐ray (oblique) and other image guidance systems, summarized in Table S6, were within 0.5 mm and 0.5° in all directions. No significant differences in the LA were observed among the head, chest, and pelvis phantoms (head–chest, *p* = 0.91; head–pelvis, *p* = 0.89; chest–pelvis, *p* = 0.66).

## 4 DISCUSSION

To the best of our knowledge, this study represents the first report to investigate the commissioning and QA of the second‐generation O‐ring‐type linear accelerator, OXRAY, with a particular emphasis on its unique noncoplanar dynamic irradiation capability, BROAD‐RT. While conventional commissioning procedures provide essential verification of the dose calculation accuracy and system performance, they are not sufficient to fully characterize the complexities introduced by the dynamic geometry of BROAD‐RT, which involves the simultaneous rotation of both the gantry and O‐ring. To address this challenge, we developed and validated a novel, EPID‐based QA methodology capable of capturing the beam trajectory with submillimeter precision under dynamic delivery conditions. The feasibility and geometric fidelity of this QA framework were further confirmed in an ablation study combining BROAD‐RT with real‐time tumor tracking, where consistently low tracking errors were observed across various respiratory‐related trajectories. The implementation of this approach not only ensures geometric fidelity during complex treatments but also offers a practical framework for future institutions adopting similar noncoplanar tracking technologies. These findings contribute to bridging the gap between standard commissioning protocols and the specialized needs of advanced delivery systems, thereby laying the groundwork for more robust and adaptive QA paradigms tailored to next‐generation radiotherapy platforms.

In this study, the median GPR value (3%/2 mm) was 97.3% for the 6 MV and 6 MV‐FFF plans (Figure 6). However, the GPR value for one of the BROAD‐RT prostate plans that used 6 MV‐FFF beams was below 90%. BROAD‐RT plans involve multiple manipulation points, which may negatively affect dosimetric robustness. A previous report has noted that at these points, variations in gantry or ring rotation speeds are observed, necessitating dose‐rate modulation as an alternative to conventional beam on/off switching.^10^ While we acknowledge that this phenomenon may influence the dose delivery accuracy, it is important to note that real‐time modulation of the dose rate in response to dynamic mechanical parameters is currently limited by the hardware architecture of the OXRAY system. Specifically, the dual‐gun configuration imposes constraints on beam control during transitions, making this modulation scheme technically challenging in the current system design. As such, this limitation should be recognized as an inherent characteristic of the device rather than an oversight in the study design. The report also observed larger MU differences between the planned and actual values around these points than those that had no manipulation points. Consistent with these findings, the present study also demonstrates that this phenomenon is particularly pronounced in FFF beams that enable a broader dose‐rate modulation range, likely contributing to the observed reduction in the pass rates. However, the results of the film dosimetry were consistent with the findings reported by Burghelea et al.^17^ This suggests that the difference in the GPR values may be attributed to the distinction between ArcCHECK, which evaluates doses near the surface of the phantom, and film dosimetry or Delta4, which assesses doses at greater depths within the phantom.

Compared with coplanar VMAT using the TrueBeam system (Varian Medical Systems, Palo Alto, CA, USA), BROAD‐RT enables noncoplanar irradiation by simultaneously rotating both the gantry and the O‐ring in various directions. This technique has been reported to improve dose distribution by reducing radiation exposure to normal tissues while maintaining adequate target coverage.^7^, ^9^ In addition, a system that allows for patient‐specific trajectory customization—beyond the pre‐implemented trajectories—has also been developed.^8^ These advanced delivery strategies all rely on the assumption that MV beam positioning accuracy is ensured. In the present study, the beam center positioning accuracy was confirmed to be within 0.4 mm, demonstrating excellent geometric performance. This result is consistent with previously reported MV beam isocenter displacements in the Vero4DRT system.^18^ Unlike conventional C‐arm linear accelerators, which are prone to gantry sagging due to the weight of the treatment head, the OXRAY system incorporates a gimbal correction mechanism that compensates for mechanical deflection, effectively minimizing sagging effects.^19^ Furthermore, OXRAY inherits the mechanical precision of its predecessor, the Vero4DRT, which has been shown to exhibit minimal positioning errors at the centers of both gantry and ring rotation.^20^ This high level of geometric accuracy underscores the importance of reliable verification methods tailored to BROAD‐RT's complex beam trajectories. To this end, we employed a QA technique based on the EPID, which is permanently mounted opposite the irradiation head in the OXRAY system. This fixed configuration enables straightforward and efficient evaluation of beam positioning accuracy and is well‐suited not only for pretreatment verification but also for routine quality assurance. Therefore, it would be reasonable to include this method in the periodic QA schedule in addition to its use during commissioning.

In this study, the tracking accuracy of the OXRAY system under rotational irradiation was found to be comparable to that previously reported for the Vero4DRT system, which demonstrated long‐term stability over a 2‐year period.^15^ The earlier investigation utilized a programmable respiratory motion phantom to simulate tumor and surrogate motion and evaluated both the predictive accuracy of the 4D correlation model and the overall tracking accuracy based on real‐time EPID images.^15^ The generated results exhibited submillimeter deviations and low coefficients of variation across all directions, confirming the system's robustness. Similarly, our results demonstrated that the tracking accuracy remained within a clinically acceptable range even under dynamic conditions involving gantry rotation, indicating that the implementation of the 4D model and real‐time correction via the gimbaled head motion in the OXRAY system is as effective as that in Vero4DRT. These findings support the clinical feasibility of OXRAY for real‐time tumor tracking during rotational beam delivery and highlight the reliability of real‐time EPID‐based assessment as a QA tool.

The LA in IGRT relies on precise and repeatable image registration and patient positioning. The repeatability and LA of image registration using the PIAS for patient positioning verification were comparable to those of other IGRT systems.^18^, ^21^, ^22^ Furthermore, no significant differences in the LA were observed among the head, chest, and pelvis phantoms. These results indicate that PIAS provides sufficient image registration accuracy, making IGRT feasible with high precision across most anatomical regions.

One limitation of this study is that certain advanced features of the OXRAY system, such as radiation field enlargement by gimbal rotation, were not evaluated as part of the commissioning process. Although this function is important, radiation field enlargement has not been implemented in the current version of the RayStation 2023B TPS and is beyond the scope of this analysis. These aspects will be addressed in future studies.

## 5 CONCLUSIONS

We conducted a simple evaluation of the OXRAY system. The beam quality of OXRAY was verified and confirmed to be suitable for clinical patient treatments, demonstrating its capability to deliver radiation doses with high precision. Moreover, even during rotational irradiation, the tracking accuracy remained within 1 mm, indicating the expected performance. In addition, the automatic image registration function provided reliable and accurate patient positioning, fulfilling the requirements for IGRT. These findings provide practical guidance and technical benchmarks for institutions planning to implement the OXRAY system.

## AUTHOR CONTRIBUTION

**Kohei Kawata**: Conceptualization; methodology; formal analysis; investigation; data curation; writing—original draft. **Yukako Kishigami**: Conceptualization; methodology; formal analysis; investigation; data curation; writing—original draft. **Hideaki Hirashima**: Software; data curation; writing—review and editing. **Yohei Sawada**: Data curation; writing—review and editing. **Maika Urago**: Data curation; writing—review and editing. **Takahiro Fujimoto**: Writing—review and editing. **Tetsuo Fukuda**: Investigation; writing—review and editing. **Takashi Mizowaki**: Writing—review and editing; funding acquisition. **Mitsuhiro Nakamura**: Conceptualization; writing—review and editing; supervision; project administration.

## CONFLICT OF INTEREST STATEMENT

Tetsuo Fukuda is an employee of Hitachi High‐Tech Corporation. Takashi Mizowaki received research grants and scholarship donations from Hitachi Ltd. Mitsuhiro Nakamura received a scholarship donation from Hitachi Ltd. The other co‐authors are Hitachi High‐Tech Corporation collaborators.

## Supporting information



Supporting Information

## Data Availability

Data supporting the findings of this study are available from the corresponding author upon request.
