# Supplementary material for: Performance evaluation of a second‐generation O‐ring‐shaped image‐guided radiotherapy system with a gimbal‐mounted linear accelerator and real‐time tracking capabilities
Source: J Appl Clin Med Phys. 2025 Nov 7;26(11):e70329. doi: 10.1002/acm2.70329 (PMC12594607; doi:10.1002/acm2.70329)
Supplement: Supplementary file 1 — Supporting Information [file ACM2-26-e70329-s001.pdf]

Supplementary Table S1. Summary of parameters for the image-guidance systems in this study. *Abbreviations*: FOV-S, field of view; CW, clockwise; CCW, counter-clockwise.

System	Imaging direction		FOV (mm)	Number of Projections	Pixel spacing (mm)	Imaging condition								
						Head			Chest			Pelvis		
						kV	mA	msec	kV	mA	msec	kV	mA	msec
kV X-ray	Oblique	315° and 45°	-	2	0.30	100	100	10	100	100	10	120	200	10
	True lateral	0° and 90°		2	0.30	100	100	10	100	100	10	120 (0°)	63 (0°)	32 (0°)
		270° and 0°	-	2	0.30	100	100	10	100	100	10	120 (90°, 270°)	400 (90°, 270°)	32 (90°, 270°)
kV-CBCT	Single (Imager 1)	200°, CW	210	391	0.428	100	100	10	90	100	5	100	320	10
		200°, CCW	210	391	0.428	100	100	10	90	100	5	100	320	10
		360°, CW	400	721	0.790	100	100	10	100	100	10	125	100	5
		360°, CCW	400	721	0.790	100	100	10	100	100	10	125	100	5
	Single (Imager 2)	200°, CW	210	391	0.428	100	100	10	90	100	5	100	320	10
		200°, CCW	210	391	0.428	100	100	10	90	100	5	100	320	10
		360°, CW	400	721	0.790	100	100	10	100	100	10	125	100	5
		360°, CCW	400	721	0.790	100	100	10	100	100	10	125	100	5
	Dual (Imager 1&2)	116°, CW	210	212	0.428	100	100	10	100	100	10	100	320	10
		116°, CCW	210	212	0.428	100	100	10	100	100	10	100	320	10

Supplementary Table S2. Summary of the largest δ_1, δ_2, δ_3, and δ_4 values for 6 MV. *Abbreviation*: *OCR*, off-center ratio. N/A: These values were not be evaluated because of the limited scan range.

Scan type	Field size (mm^2)	Depth (mm)	δ_1 (%)	δ_2 (mm)	δ_3 (%)	δ_4 (%)
Absolute depth dose	20 × 20	-	0.7	-0.5	-	-
	50 × 50	-	0.8	-0.7	-	-
	100 × 100	-	0.5	-0.5	-	-
	200 × 200	-	-0.3	0.0	-	-
OCR (Crossline)	20 × 20	15	-	-0.1	0.2	-0.2
		100	-	-0.2	1.1	-1.3
		200	-	-0.3	1.4	-2.3
	50 × 50	15	-	-0.1	-0.2	0.3
		100	-	-0.1	0.3	0.1
		200	-	-0.1	0.7	-0.6
	100 × 100	15	-	-0.1	-0.3	0.6
		100	-	-0.1	0.0	-0.3
		200	-	-0.1	-0.3	N/A
	200 × 200	15	-	0.0	0.5	N/A
		100	-	0.0	0.2	N/A
		200	-	-0.1	-0.1	N/A
OCR (Inline)	20 × 20	15	-	0.0	0.3	-0.2
		100	-	0.0	1.0	-1.1
		200	-	-0.1	1.1	-2.1
	50 × 50	15	-	0.0	-0.1	0.5
		100	-	0.0	0.5	0.3
		200	-	-0.1	0.8	-0.1
	100 × 100	15	-	-0.1	-0.1	0.8
		100	-	-0.1	0.2	0.1
		200	-	-0.2	-0.1	N/A
	200 × 200	15	-	0.0	0.5	N/A
		100	-	0.0	0.2	N/A
		200	-	-0.1	-0.3	N/A

Supplementary Table S3. Summary of the largest δ_1, δ_2, δ_3, and δ_4 values for 6 MV-FFF. *Abbreviation*: *OCR*, off-center ratio. N/A: These values were not be evaluated because of the limited scan range.

Scan type	Field size (mm^2)	Depth (mm)	δ_1 (%)	δ_2 (mm)	δ_3 (%)	δ_4 (%)
Absolute depth dose	20 × 20	-	0.4	-0.8	-	-
	50 × 50	-	0.6	-1.1	-	-
	100 × 100	-	-0.4	-0.9	-	-
	200 × 200	-	0.4	-0.6	-	-
OCR (Crossline)	20 × 20	15	-	-0.1	0.0	-0.3
		100	-	-0.2	0.9	-0.8
		200	-	-0.3	0.3	-1.4
	50 × 50	15	-	0.0	0.1	0.2
		100	-	0.0	0.3	-0.2
		200	-	-0.1	0.5	-0.9
	100 × 100	15	-	0.0	-0.3	0.3
		100	-	0.0	0.1	-0.4
		200	-	-0.1	-0.3	N/A
	200 × 200	15	-	0.1	0.3	N/A
		100	-	-0.1	0.0	N/A
		200	-	-0.1	0.0	N/A
OCR (Inline)	20 × 20	15	-	0.0	0.4	-0.2
		100	-	0.0	1.2	-0.7
		200	-	-0.1	-0.1	-1.1
	50 × 50	15	-	0.0	0.0	0.3
		100	-	0.0	0.2	0.0
		200	-	-0.1	0.3	-0.8
	100 × 100	15	-	0.0	-0.1	0.5
		100	-	-0.1	0.1	-0.2
		200	-	-0.1	0.2	N/A
	200 × 200	15	-	0.2	0.3	N/A
		100	-	0.2	0.3	N/A
		200	-	0.1	-0.5	N/A

Supplementary Table S4. Summary of the deliverable manipulation points for trajectories of BROAD-RT.

Trajectory	Deliverable manipulation points [Gantry/Ring (degree)]								
	1	2	3	4	5	6	7	8	9
C1 Cranial	182/0	238/25	322/325	38/35	122/335	178/0	-	-	
C2 Cranial	182/0	310/40	50/320	178/0	-	-	-	-	
C3 Cranial	182/0	290/40	70/320	178/0	-	-	-	-	
C4 Cranial	182/0	242/40	346/340	54/320	178/0	-	-	-	
C5 Cranial	182/0	306/40	14/20	118/320	178/0	-	-	-	
C6 Cranial (L)	182/340	260/345	310/320	40/320	100/345	178/320	-	-	
C6 Cranial (R)	182/20	260/15	310/40	40/40	100/15	178/20	-	-	
E1 ExtraCranial	182/0	290/15	70/345	178/0	-	-		-	
B1 Breast A (L)	300/340	320/20	348/34	60/20	120/340	140/20	-	-	
B2 Breast A (R)	220/340	240/20	300/340	12/326	40/340	60/20	-	-	
B3 Breast B (L)	300/340	320/20	348/34	60/20	120/340	140/20	-	-	
B4 Breast B (R)	288/16	308/336	50/30	100/1	140/340	160/20	-	-	
L1 Liver	182/20	270/10	310/355	2/0	50/15	90/0	178/15	-	-
L2 Liver	182/0	270/10	310/30	50/330	90/10	178/0	-	-	-
L3 Lung	182/20	234/0	314/25	42/335	90/5	178/350	-	-	-
L5 Lung	182/340	234/0	314/335	42/25	178/340	-	-	-	-
P1 Pancreas	182/20	234/0	314/25	42/335	100/10	178/340	-	-	-
P2 Pancreas	182/20	234/0	314/335	42/25	178/340	-	-	-	-
P3 Pancreas	182/20	250/0	314/25	42/335	90/5	178/350	-	-	-
P3 Prostate	182/0	230/0	282/20	310/340	50/20	78/340	130/0	178/0	-

P4 Prostate	182/10	230/0	282/20	310/340	50/20	78/340	130/0	178/350	-
X-Arc1	182/0	298/20	62/32	178/0	-	-	-	-	-
X-Arc2	182/0	298/328	62/340	178/0	-	-	-	-	-
X1 Generic	182/10	228/350	272/10	316/350	0/10	44/350	88/10	132/350	178/10
X2 Generic	182/0	228/345	316/15	44/345	132/15	178/0	-	-	-

Supplementary Table S5. Localization accuracy using a patient positioning image analysis system for all image guidance systems and anthropomorphic phantoms. *Abbreviations*: CW, clockwise; CCW, counter-clockwise; FOV-S, field of view with standard (210 mm in diameter); FOV-L, field of view with large (400 mm in diameter).

Phantom	Image guidance system	Image direction		Translation deviation (mm)			Rotational deviation (°)		
				Lateral	Longitudinal	Vertical	Roll	Pitch	Ring
Head	X-ray	Oblique	315° and 45°	-0.1±0.1	0.0±0.0	0.0±0.1	0.0±0.0	-0.1±0.1	0.0±0.1
		True lateral	0° and 90°	0.0±0.1	0.0±0.1	0.0±0.1	0.2±0.1	0.1±0.1	0.1±0.1
			270° and 0°	-0.1±0.2	0.0±0.0	-0.3±0.1	0.0±0.3	-0.2±0.1	0.0±0.0
	CBCT	Imager 1	CW (FOV-S)	-0.1±0.1	-0.1±0.1	0.1±0.0	0.1±0.1	0.1±0.1	0.0±0.0
			CCW (FOV-S)	0.0±0.1	0.0±0.0	0.1±0.1	0.0±0.1	0.1±0.0	0.0±0.0
			CW (FOV-L)	-0.1±0.1	0.0±0.1	0.0±0.1	-0.2±0.1	-0.1±0.1	0.0±0.1
			CCW (FOV-L)	0.0±0.1	0.0±0.1	0.0±0.1	0.0±0.2	0.0±0.1	0.0±0.1
		Imager 2	CW (FOV-S)	-0.1±0.1	-0.1±0.0	0.1±0.0	0.0±0.1	0.1±0.0	0.0±0.0
			CCW (FOV-S)	0.0±0.1	-0.1±0.1	0.1±0.1	0.1±0.1	0.1±0.0	0.0±0.1
			CW (FOV-L)	0.1±0.1	0.0±0.1	0.1±0.1	0.4±0.2	0.0±0.0	0.0±0.0
			CCW (FOV-L)	0.1±0.1	-0.1±0.1	0.0±0.1	0.4±0.2	0.0±0.0	0.0±0.0
		Imager 1 and 2	CW (FOV-S)	0.0±0.1	0.0±0.1	0.1±0.0	0.0±0.1	0.1±0.0	0.0±0.0
			CCW (FOV-S)	0.0±0.1	-0.1±0.1	0.0±0.1	0.1±0.1	0.0±0.0	0.0±0.0
Chest	X-ray	Oblique	315° and 45°	0.0±0.0	0.0±0.0	-0.1±0.0	0.0±0.0	0.0±0.1	0.0±0.0
		True lateral	0° and 90°	-0.1±0.1	0.0±0.1	-0.1±0.1	0.0±0.1	0.0±0.0	0.0±0.0
			270° and 0°	0.0±0.1	0.1±0.1	-0.1±0.1	0.0±0.1	0.1±0.0	0.0±0.0
	CBCT	Imager 1	CW (FOV-S)	0.0±0.1	0.2±0.1	0.0±0.1	-0.1±0.1	0.0±0.0	0.0±0.1
			CCW (FOV-S)	0.0±0.1	0.1±0.1	0.0±0.0	0.0±0.1	0.0±0.1	0.0±0.1

			CW (FOV-L)	0.0±0.1	0.1±0.1	-0.1±0.1	0.1±0.1	0.0±0.1	0.0±0.1
			CCW (FOV-L)	-0.1±0.2	-0.1±0.1	0.0±0.1	0.2±0.1	0.0±0.1	0.0±0.1
		Imager 2	CW (FOV-S)	0.0±0.1	0.1±0.1	-0.1±0.0	-0.1±0.1	0.0±0.1	0.0±0.1
			CCW (FOV-S)	0.0±0.1	-0.1±0.1	0.0±0.0	0.1±0.1	0.0±0.0	0.0±0.0
			CW (FOV-L)	0.0±0.1	0.1±0.1	0.0±0.1	0.0±0.0	0.0±0.1	0.0±0.0
			CCW (FOV-L)	0.1±0.1	0.0±0.1	0.0±0.0	-0.1±0.1	0.0±0.0	0.1±0.0
		Imager 1 and 2	CW (FOV-S)	0.1±0.1	0.1±0.1	0.0±0.1	0.2±0.3	0.0±0.0	0.0±0.0
			CCW (FOV-S)	0.0±0.1	0.0±0.1	-0.1±0.1	-0.1±0.1	0.1±0.1	0.0±0.0
Pelvis	X-ray	Oblique	315° and 45°	0.0±0.0	0.0±0.1	0.0±0.0	0.0±0.1	0.0±0.1	0.0±0.0
		True lateral	0° and 90°	-0.1±0.0	0.0±0.1	0.0±0.0	0.0±0.1	0.0±0.1	0.0±0.0
			270° and 0°	-0.1±0.0	0.0±0.0	-0.1±0.1	0.0±0.1	0.1±0.0	0.0±0.0
	CBCT	Imager 1	CW (FOV-S)	0.1±0.1	0.1±0.1	0.0±0.0	-0.1±0.1	0.2±0.1	-0.1±0.1
			CCW (FOV-S)	-0.1±0.1	0.1±0.2	0.0±0.1	0.0±0.0	0.0±0.1	0.0±0.1
			CW (FOV-L)	0.0±0.0	0.3±0.4	-0.1±0.1	0.1±0.0	0.0±0.1	-0.1±0.1
			CCW (FOV-L)	0.0±0.1	0.3±0.3	0.0±0.1	0.1±0.0	0.0±0.1	0.0±0.1
		Imager 2	CW (FOV-S)	-0.1±0.2	0.1±0.1	0.1±0.1	-0.1±0.1	0.0±0.1	0.0±0.1
			CCW (FOV-S)	0.1±0.1	0.1±0.1	0.0±0.1	0.1±0.1	0.2±0.1	0.0±0.1
			CW (FOV-L)	0.1±0.1	0.2±0.1	0.0±0.1	0.0±0.1	-0.1±0.0	0.0±0.1
			CCW (FOV-L)	0.1±0.1	0.3±0.2	-0.1±0.1	0.0±0.1	0.0±0.1	0.0±0.1
		Imager 1 and 2	CW (FOV-S)	-0.1±0.2	0.1±0.1	0.0±0.1	0.0±0.1	0.1±0.0	0.0±0.1
			CCW (FOV-S)	-0.1±0.2	0.2±0.2	0.0±0.2	0.0±0.2	0.0±0.2	0.0±0.0

Supplementary Table S6. Differences in fusion results between kV X-ray (oblique) and other image-guidance systems. *Abbreviations*: CW, clockwise; CCW, counter-clockwise; FOV-S, field of view with standard (210 mm in diameter); FOV-L, field of view with large (400 mm in diameter).

Phantom	Image guidance system	Image direction		Translation deviation (mm)			Rotational deviation (°)		
				Lateral	Longitudinal	Vertical	Roll	Pitch	Ring
Head	X-ray	Oblique	315° and 45°	-	-	-	-	-	-
		True lateral	0° and 90°	0.0±0.1	-0.1±0.1	0.0±0.1	-0.3±0.1	0.1±0.1	0.0±0.1
			270° and 0°	-0.1±0.2	-0.1±0.1	-0.1±0.1	-0.4±0.2	0.0±0.1	0.0±0.2
	CBCT	Imager 1	CW (FOV-S)	-0.1±0.0	-0.1±0.1	0.1±0.1	0.0±0.1	0.0±0.1	-0.2±0.1
			CCW (FOV-S)	-0.2±0.1	-0.1±0.1	-0.1±0.0	0.0±0.1	0.1±0.0	-0.2±0.1
			CW (FOV-L)	-0.1±0.0	-0.1±0.1	0.0±0.2	0.0±0.1	0.0±0.1	-0.2±0.0
			CCW (FOV-L)	-0.1±0.1	-0.1±0.1	0.0±0.1	0.1±0.1	-0.1±0.1	-0.2±0.1
		Imager 2	CW (FOV-S)	-0.2±0.1	-0.2±0.1	-0.1±0.0	-0.1±0.1	0.1±0.0	-0.2±0.1
			CCW (FOV-S)	0.0±0.1	-0.2±0.0	-0.2±0.0	-0.1±0.1	-0.1±0.0	-0.2±0.1
			CW (FOV-L)	-0.1±0.1	-0.1±0.1	0.0±0.1	0.2±0.3	-0.1±0.1	-0.2±0.1
			CCW (FOV-L)	-0.1±0.1	-0.1±0.1	-0.1±0.2	0.2±0.3	-0.1±0.1	-0.2±0.1
		Imager 1 and 2	CW (FOV-S)	-0.2±0.0	-0.1±0.1	-0.1±0.0	0.1±0.0	0.0±0.1	-0.2±0.1
			CCW (FOV-S)	-0.2±0.0	-0.1±0.1	-0.2±0.0	-0.1±0.1	0.0±0.0	-0.2±0.1
Chest	X-ray	Oblique	315° and 45°	-	-	-	-	-	-
		True lateral	0° and 90°	-0.3±0.1	-0.1±0.0	-0.1±0.1	0.2±0.1	0.0±0.0	0.0±0.0
			270° and 0°	0.0±0.1	0.0±0.0	0.0±0.1	0.0±0.1	0.1±0.0	0.0±0.0
	CBCT	Imager 1	CW (FOV-S)	0.1±0.0	0.1±0.0	0.2±0.0	0.1±0.0	-0.1±0.0	-0.1±0.0
			CCW (FOV-S)	-0.1±0.0	0.0±0.1	0.0±0.0	0.1±0.0	-0.1±0.1	-0.2±0.0
			CW (FOV-L)	0.2±0.1	0.0±0.1	-0.2±0.1	0.0±0.1	0.0±0.0	-0.2±0.0

			CCW (FOV-L)	0.1±0.1	0.0±0.1	-0.1±0.1	0.1±0.1	0.0±0.1	-0.2±0.1
		Imager 2	CW (FOV-S)	-0.1±0.0	-0.1±0.0	0.1±0.0	0.0±0.1	0.0±0.1	-0.1±0.1
			CCW (FOV-S)	0.0±0.0	-0.1±0.1	0.0±0.1	0.0±0.1	-0.1±0.0	-0.2±0.0
			CW (FOV-L)	0.2±0.1	0.2±0.1	0.0±0.1	0.0±0.1	0.1±0.0	-0.1±0.0
			CCW (FOV-L)	0.2±0.1	0.2±0.1	-0.1±0.0	0.0±0.1	0.1±0.0	-0.1±0.1
		Imager 1 and 2	CW (FOV-S)	-0.1±0.1	0.0±0.0	0.2±0.1	0.2±0.3	-0.1±0.1	-0.2±0.0
			CCW (FOV-S)	0.0±0.1	-0.1±0.1	0.0±0.1	-0.1±0.1	-0.1±0.1	-0.2±0.0
Pelvis	X-ray	Oblique	315° and 45°	-	-	-	-	-	-
		True lateral	0° and 90°	0.0±0.0	0.0±0.0	0.2±0.0	0.2±0.0	0.1±0.1	0.0±0.0
			270° and 0°	-0.1±0.0	0.1±0.0	0.1±0.1	-0.1±0.1	0.1±0.1	-0.1±0.0
	CBCT	Imager 1	CW (FOV-S)	0.1±0.1	-0.2±0.1	0.3±0.1	0.0±0.1	0.1±0.1	-0.1±0.1
			CCW (FOV-S)	-0.1±0.1	-0.1±0.2	-0.3±0.0	0.2±0.0	0.3±0.1	0.0±0.1
			CW (FOV-L)	0.1±0.0	-0.3±0.3	-0.3±0.1	0.1±0.0	-0.1±0.1	-0.1±0.1
			CCW (FOV-L)	0.1±0.0	-0.4±0.3	-0.2±0.0	0.1±0.0	-0.1±0.1	-0.1±0.1
		Imager 2	CW (FOV-S)	-0.3±0.1	-0.2±0.1	-0.2±0.1	0.0±0.0	0.3±0.0	-0.1±0.1
			CCW (FOV-S)	0.0±0.2	-0.3±0.1	0.0±0.1	0.0±0.1	0.1±0.2	0.0±0.0
			CW (FOV-L)	-0.1±0.0	-0.5±0.2	-0.3±0.0	0.5±0.1	0.0±0.1	-0.1±0.1
			CCW (FOV-L)	-0.1±0.0	-0.4±0.2	-0.3±0.1	0.0±0.0	0.0±0.1	-0.1±0.1
		Imager 1 and 2	CW (FOV-S)	-0.3±0.2	-0.2±0.1	0.0±0.1	0.0±0.1	0.1±0.1	0.0±0.1
			CCW (FOV-S)	-0.1±0.1	-0.2±0.2	-0.2±0.1	0.2±0.0	0.1±0.1	0.0±0.0
